# Human papillomavirus type 18 oncoproteins exert their oncogenicity in esophageal and tongue squamous cell carcinoma cell lines distinctly

**DOI:** 10.1186/s12885-019-6413-7

**Published:** 2019-12-12

**Authors:** Siaw Shi Boon, Zigui Chen, Jintao Li, Karen Y. C. Lee, Liuyang Cai, Rugang Zhong, Paul K. S. Chan

**Affiliations:** 1 Department of Microbiology, Faculty of Medicine, The Chinese University of Hong Kong, Shatin, Hong Kong; 2 Beijing Key Laboratory of Environmental and Viral Oncology, College of Life Science and Bio-engineering, Beijing University of Technology, Beijing, China

**Keywords:** HPV18, Esophageal squamous cell carcinoma, Tongue squamous cell carcinoma, E6, E7

## Abstract

**Background:**

Increasing evidence indicates an etiological role of human papillomavirus (HPV) in head and neck cancers, particularly oropharyngeal squamous cell carcinoma (OPSCC). However, the association between HPV and other cancers, including esophageal and tongue remains unclear. This study delineated the molecular characteristics of HPV18 E6 and E7 in esophageal (EC109 and EC9706) and tongue (Tca83) cancer cell lines with reference to cervical cancer (HeLa).

**Methods:**

We analysed the HPV transcription profiles of esophageal and tongue cancer cells through Next-generation RNA sequencing, and the role of HPV18 E6 and E7 in these cells was assessed via siRNA approach, Western blotting and immunofluorescence assays.

**Results:**

Overall, the HPV transcription profiles of esophageal and tongue cancer cells mimicked that of cervical cancer cells, with notable disruption of E2, and expression of E6, spliced E6 (E6^*^), E7, E1 and L1 transcripts. As with cervical cancer cells, p53 and its downstream transactivation target, p21, were found to be the major targets of E6 in esophageal and tongue cancer cell lines. Intriguingly, E7 preferentially targeted p130 in the two esophageal cancer cell lines, instead of pRb as in cervical cancer. Tca83 exhibited an E7 to E6 transcript ratio comparable to HeLa (cervix), targeted the ERK1/2 and MMP2 pathways, and was dependent on E6 and E7 to survive and proliferate. In contrast, both the esophageal cancer cell lines were distinct from HeLa in these aspects.

**Conclusions:**

This is the first study that delineates transcript expression and protein interaction of HPV18 E6 and E7 in esophageal and tongue cancer cell lines, suggesting that HPV plays a role in inducing these cancers, albeit via distinct pathways than those observed in cervical cancer.

## Background

Head and neck cancers (HNC) and esophageal cancers (EC) are ranked the seventh and sixth most common causes of cancer death worldwide, respectively [1]. HNC incidence poses a worrisome increment in many geographical regions. It was estimated that the incidence of oropharyngeal cancers might further increase in the United States and European countries [2–4]. Meanwhile, esophageal squamous cell carcinoma is highly prevalent in the so-called “esophageal cancer belt”, including Northern Iran, Central Asia, North-Central China, along the Rift Valley in East Africa, and South Africa [5]. Among these countries, China is the most affected, particularly in rural areas such as the Henan province [5].

Both HNC and EC appear to share similar risk factors, including poverty, alcohol and tobacco consumption [6, 7], diet and nutrition [8, 9], as well as exposure to environmental carcinogens such as polycyclic aromatic hydrocarbons (PAHs) [10, 11]. Human papillomavirus (HPV), mainly HPV16 followed by HPV18, is now recognized as a cause of a fraction of oropharyngeal cancers [12–14]. However, the etiological role of HPV in tongue and esophageal cancers is still controversial [15–18]. HPV-induced carcinogenesis is mainly driven by the viral oncoproteins, E6 and E7, which are essential in maintaining tumor phenotype. E6 and E7 are multi-functional proteins involved in several cellular processes, including caspase-mediated apoptosis, cell cycle progression and signaling pathways. E6 mediates downregulation of p53 [19–21] and PSD95/Dlg/ZO-1 (PDZ) proteins [22–24], leading to perturbed p21 functions and cell polarity, respectively. Whilst E7 downregulates retinoblastoma protein (pRB) and its related pocket proteins, including p130 and p107 [25–27], leading to transactivation of E2F response promoter genes [28]. In addition, HPV oncoproteins can deregulate AKT [29], ERK [30] and metalloproteases (MMPs) [31, 32], leading to cancer formation and progression. All these are classical molecular targets of HPV oncoproteins in cervical cancer.

Observations at molecular level from established cancer cell lines could improve our understanding on the role of HPV in esophageal and tongue squamous cell carcinoma (SCC). In this study, we analyzed the transcript expression profiles and functions of E6 and E7 to delineate the role of HPV18 in esophageal (EC109 and EC9706) and tongue (Tca83) cancers based on cell lines established from Chinese.

## Methods

### Cell lines

Esophageal squamous cell carcinoma (SCC) (EC109 and EC9706) and tongue SCC (Tca83) cell lines were derived from patients in China. We included HeLa cells (HPV18-positive) originally derived from cervical cancer, and HKESC01 (HPV-null) from an esophageal cancer patient of Chinese origin, as references. HeLa cell line was purchased from the American Type Culture Collection (ATCC). The EC109, EC9706 and Tca83 cell lines were generous gifts from Prof. Zeng Yi, the National Institute for Viral Disease Control and Prevention of Chinese Center for Disease Control and Prevention in 2012. The HKESC01 cell line was a generous gift from Prof. Chi Hin Cho, the Chinese University of Hong Kong in 2017. All these cell lines have been authenticated using Short Tandem Repeat (STR) profiling this year. These mycoplasma-free cells were maintained in Dulbecco’s Modified Eagle Medium (DMEM), supplemented with 10% FBS in a 37 °C humidified incubator containing 5% CO_2_.

### Next-generation RNA sequencing and bioinformatics analysis

Total RNA of each cell line was extracted using RNeasy Mini Kit (Qiagen), treated with DNase, and prepared for Next-generation sequencing library using TruSeq Stranded Total RNA LT (Illumina), according to the manufacturer’s instructions. Briefly, libraries were run on an Illumina HiSeq4000 for paired-end 100 bp sequencing. The RNA-seq data were analyzed according to the HISAT2-StringTie-Ballgown pipeline [33]. In brief, high-quality reads filtered by Trimmomatic V0.38 were mapped to the human genome index (GRCh38) using HISAT2 v2.1.0 with default parameters. A read coverage table was generated using StringTie v1.3.5 against a GRCh38 gtf annotation file, following the normalization procedure using Fragments per kilobase of transcript per million reads mapped (FPKM). We also built a HISAT2 transcript index and a gtf annotation file for HPV18 [34]. The R package Ballgown was used to create differential expression tables and plot gene transcript patterns. The RNA sequence data have been deposited in the NCBI Gene Expression Omnibus database (GEO, http://www.ncbi.nlm.nih.gov/geo/) and are accessible through GEO Series accession number (SRA Accession: PRJNA530677).

### Downregulation of HPV18 E6 and E7

Approximately 2 × 10^5^ of all the cells were seeded into 6-well plates. After 24 h, HPV18 E6 and E7 expression were ablated by transfecting the cells with small interfering RNA (siRNA) against HPV18 E6/E7 (5’CAU UUA CCA GCC CGA CGA G) (siE6/E7) using Lipofectamine LTX reagent (Invitrogen) for 72 h, according to protocol recommended by the manufacturer. Simultaneously, an independent set of cells were transfected with siControl (Dharmacon) to serve as a non-targeting siRNA negative control.

### Western blotting

Total cell extracts were obtained by lysing the cells directly using 2× SDS-PAGE sample buffer. Western blotting and processing were then performed as described previously [35]. The following antibodies were used: mouse monoclonal anti-human pRB (BD Pharmingen); rabbit polyclonal anti-MMP2 and MMP9 (Abcam); rabbit monoclonal p21 (12D1), rabbit polyclonal phospho-Akt (Ser473), rabbit monoclonal pan Akt (Cell Signaling), mouse monoclonal anti-p53 (DO-1), mouse monoclonal anti-β-actin, mouse monoclonal anti-SAP97 (2D11) (Dlg), rabbit polyclonal p130 (C-20) and p107 (C-18), mouse monoclonal p-ERK1/2 (12D4), and ERK1/2 (C-9) were from Santa Cruz.

Immunoblots were developed using Clarity™ Western ECL Substrate (Bio-Rad) and images were captured using the ChemiDoc™ Imaging System (Bio-Rad). Protein band intensities were quantified using ImageJ and normalized with the levels of β-actin, which serves as a loading control.

### Immunofluorescence

Approximately 2 × 10^5^ cells were plated onto coverslips. After overnight incubation, cells were transfected with siRNA against HPV18 E6 and E7 (siE6/E7) or siControl, as described above. After 72 h, cells were fixed with ice-cold absolute methanol. Cells were then incubated with specific primary antibodies against Ki67 (Santa Cruz) and p53 (Cell signaling), followed by relevant Alexa Fluor®568-conjugated anti-rabbit and Alexa Fluor®488-conjugated anti-mouse secondary antibodies (ThermoFisher Scientific), and counterstained with 4′,6-diamidino-2-phenylindole (DAPI). Cells were examined under a fluorescence microscope (Leica).

### Data availability statement

As mentioned above, the RNA sequence data is accessible through GEO Series accession number (SRA Accession: PRJNA530677). Data can be made available upon request.

## Results

### Esophageal and tongue cancer cell lines shared similar expression profile with cervical cancer cells, but exhibited different E7/E6 ratios

**Fig. 1:**
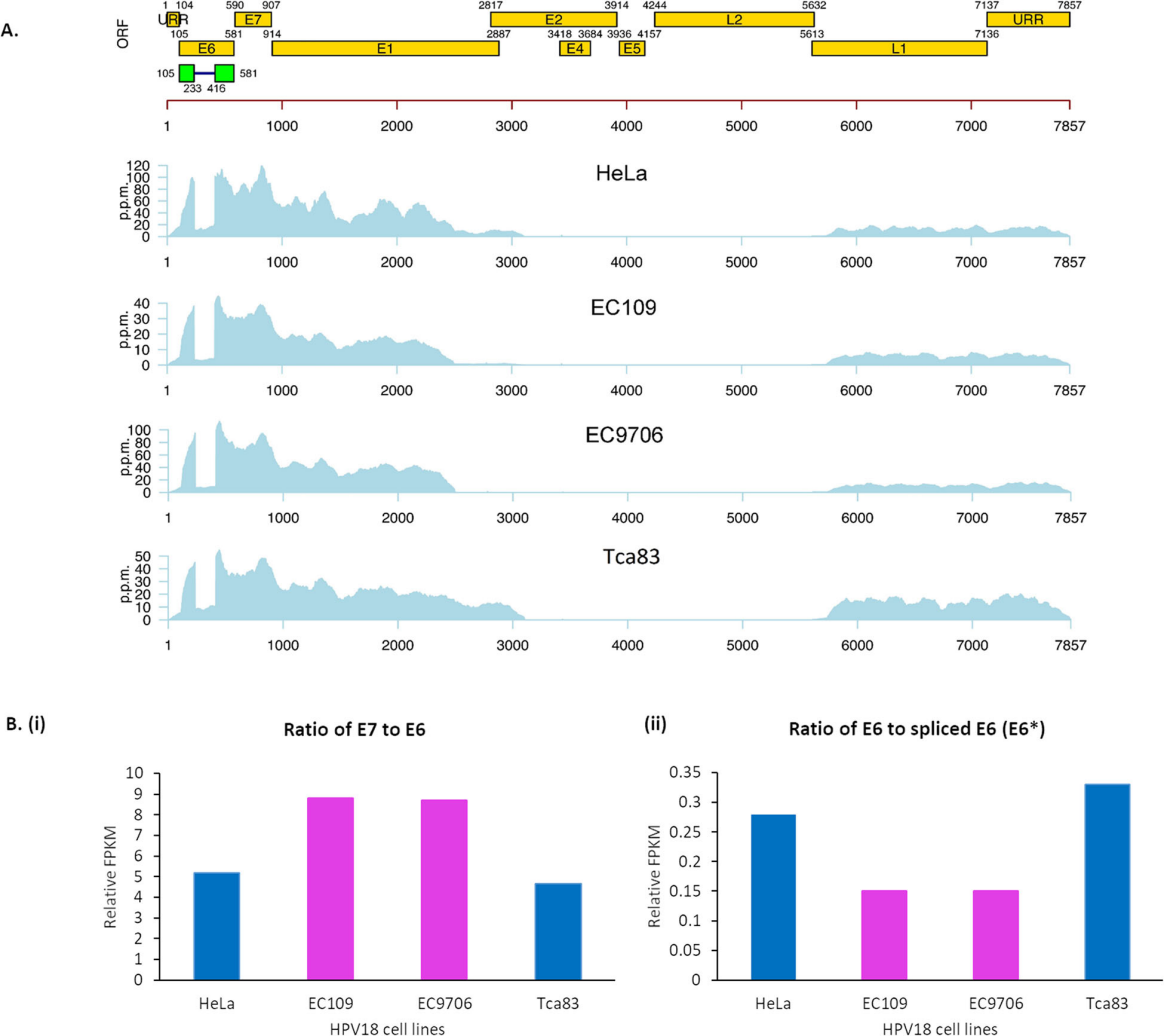
Transcription profiles of the HPV18 genome in EC109, EC9706 and Tca83 cell lines measured through RNA sequencing. (**a**) Map of HPV genome showing protein-coding regions and their respective genomic locations. (**b**) Bar charts showing relative ratios of Fragments per kilobase of transcript per million reads mapped (FPKM) of (**i**) E7 to E6, and (**ii**) E6 to spliced E6 isoform I (E6^*^)

Viral genome integration resulting in disruption and loss of viral transcripts are remarkable features of HPV-mediated oncogenesis. Therefore, we examined the HPV transcription profiles in esophageal (EC109 and EC9706), tongue (Tca83) and cervical (HeLa) cancer cell lines. Relative abundance of HPV transcripts was presented in parts per million (ppm). Overall, all these cell lines expressed E6, spliced E6 (E6*), E7, E1 and L1 transcripts (Fig. 1a). However, we noted that E1 transcripts were partially expressed in both EC109 and EC9706. Other HPV transcripts (E2, E4, E5, E8 and L2) were not detected in all the cell lines. These HPV genome profiling results were consistent with previous reports [36, 37].

Although relative abundance of transcripts originating from actively expressed regions of the viral genome were similar among these cell lines, differences in E6 and E7 transcript levels among cells were noted based on FPKM values. HeLa cells exhibited the highest level of HPV18 E6 transcripts (115,690), followed by Tca83 (98,246), EC9706 (71,897) and EC109 (70,874) (Table 1). Whilst spliced E6 variant I (E6*I) and E7 were markedly higher in EC109, EC9706 and HeLa (E6*I: 412,299 - 491,899; E7: 599,610 - 626,397) compared to Tca83 (E6*I: 293,362; E7: 457,654) (Table 1). Furthermore, the E7 to E6 ratios in EC109 and EC9706 were nearly doubled relative to those in HeLa and Tca83 (Fig. 1b), whereas HeLa and Tca83 showed nearly doubled E6:E6*I ratios compared to EC109 and EC9706. Overall, these data revealed that while HPV18 genomes exhibited similar expression profiles in the esophageal, tongue and cervical cell lines examined, subtle differences in E6 and E7 expression patterns were noted.

### HPV18 E6 in EC109, EC9706 and Tca83 targets p53 and its downstream targets in a similar manner

Following the differential expression of HPV18 oncoproteins reported above, we next examined whether E6 and E7 oncoproteins in esophageal (EC109 and EC9706) and tongue (Tca83) cancer cells target key cellular proteins in a similar manner to cervical cancer cells, such as HeLa. The cells were transfected with siRNA against HPV18 E6 and E7 (si18E6/E7). After 72 h, total protein was extracted and the levels of proteins targeted by E6, including p53, p21 and hDlg, were analyzed via Western blotting.

**Fig. 2:**
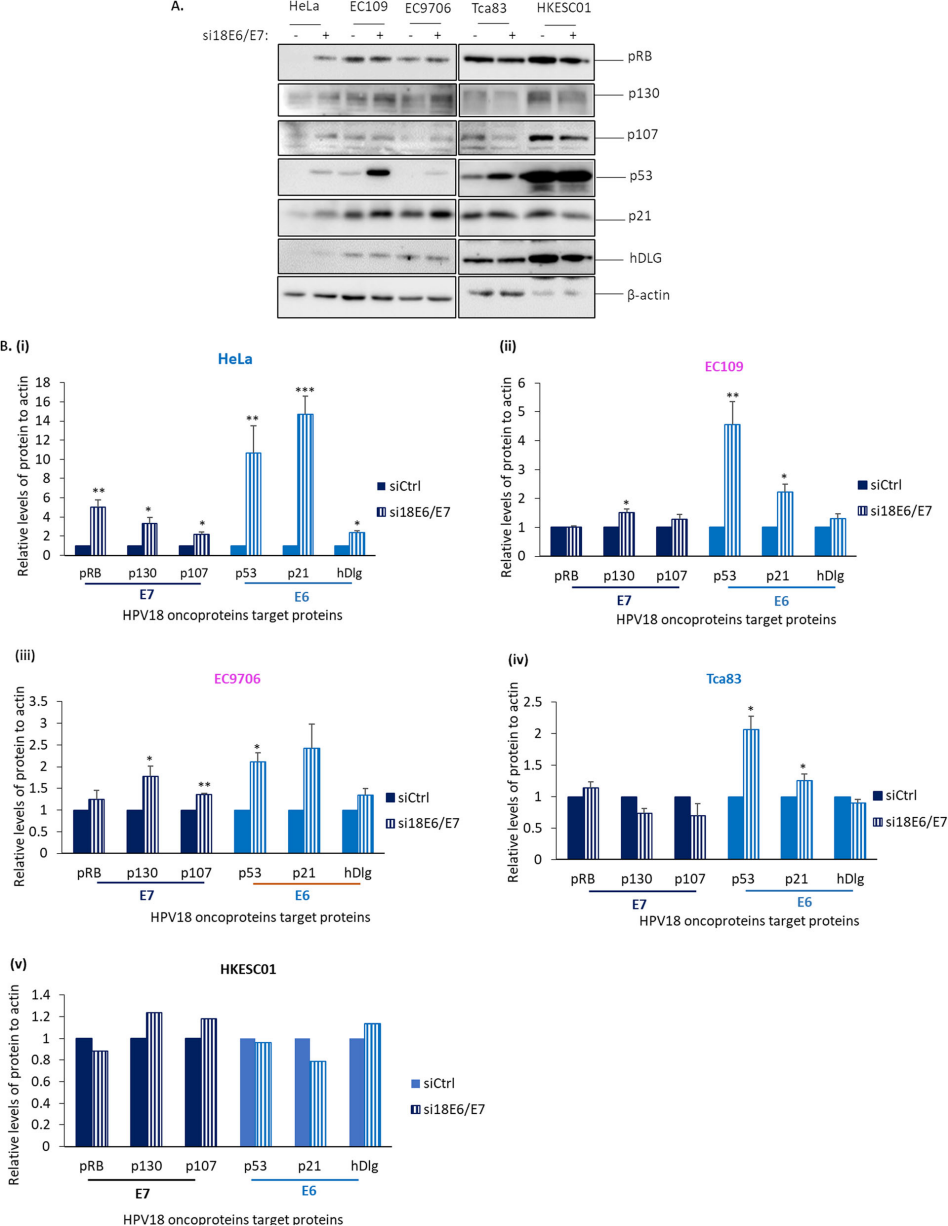
Effects of downregulation of HPV18 E6 and E7 on p53, pRB and its related pocket proteins in EC109, EC9706 and Tca83 cells. These cells were transfected with siRNA against control (−) or against HPV18 E6 and E7 (+). After 72 h, total lysate was collected and the levels of E6 and E7 target proteins were analyzed by Western blotting for the proteins as indicated. HeLa and HKESC01 cells were included as positive and negative controls, respectively. **a.** Representative immunoblots showing levels of E7 (pRB, p103 and p107) (navy blue-colored bars) and E6 (p53, p21 and hDlg) (light blue-colored bars) target proteins. β-actin was included as a loading control. **b.** Bar graphs show quantitation of the levels of target proteins against control (siCtrl) in (i) HeLa, (ii) EC109, (iii) EC9706, (iv) Tca83 and (v) HKESC01 cells. Quantitation was performed using ImageJ software and statistical analysis was performed using Prism. Error bars represent mean ± standard deviation (SD) (*n* = 4). (*P < 0.05, **P < 0.01, ***P < 0.001)

We found that HPV18 E6 in all tested cell lines behaved similarly in perturbing its major target, p53, but not PDZ protein. We found that, like HeLa [Fig. 2a and b (i)], downregulation of HPV18 E6 in all the esophageal (EC109 and EC9706) and tongue (Tca83) SCC cell lines resulted in a significant rescue of p53 as well as its downstream transactivation target, p21 [Fig. 2a and b (ii, iii and iv)]. In addition, we observed increased levels of hDlg (a PDZ protein), in HeLa cells [Fig. 2a and b (i)] upon depletion of E6, but not in the esophageal and tongue SCC cell lines examined [Fig. 2a and b (ii, iii and iv)].

### pRB is not the major target of HPV E7 in EC109, EC9706 and Tca83

As expected, we observed that downregulation of HPV18 E6 and E7 oncoproteins led to rescue of E7 targets (pRB, p130 and p107) in HeLa cells [Fig. 2a and b (ii, iii, iv)]. However, there was no significant change in the levels of pRB when E7 was downregulated in the esophageal (EC109 and EC9706) and tongue (Tca83) cell lines [Fig. 2a and b (ii, iii, iv)]. We observed significantly increased levels of p130 in both EC109 [Fig. 2a and b (ii)] and EC9706 [Fig. 2a and b (iii)], and increased p107 was only found in EC9706 [Fig. 2a and b (iii)]. Furthermore, downregulation of E7 in Tca83 did not affect the levels of pRB and its related pocket proteins [Fig. 2a and b (iv)].

### RB1, RB2 and p53 transcripts were not mutated in EC109, EC9706 and Tca83

As we found that downregulation of HPV18 E6 and E7 had no effect on the E7 major target protein, pRB, in esophageal (EC109 and EC9706) and tongue (Tca83) cell lines, we further analyzed our RNA-seq data to look at FPKM values of RB1 (encoding for pRB), RB2 (encoding for p130) and TP53 (encoding for p53) transcripts in HeLa, EC109, EC9706 and Tca83. As shown in Table 1, expression of RB1, RB2 and TP53 in all these cell lines were comparable for all these HPV-positive cells.

We further examined whether these transcripts harbored mutations that could potentially lead to amino acid alterations, and subsequently affect E7-pRB recognition in EC109, EC9706 and Tca83 compared to HeLa. We observed that RB2 harbored same-sense mutations, corresponding to amino acid positions at T694, R679 and T864, while no exonic mutation was detected within RB1 (Additional file 1). On the other hand, we found all the cell lines carried the most common TP53 polymorphism converting Proline at amino acid codon 72 to Arginine (P72R) (Additional file 1), which is consistent with previous reports [38–40].

### Tca83 cells, but not EC109 and EC9706, resemble HeLa cells in targeting ERK1/2 and MMP2 signaling pathways

**Fig. 3:**
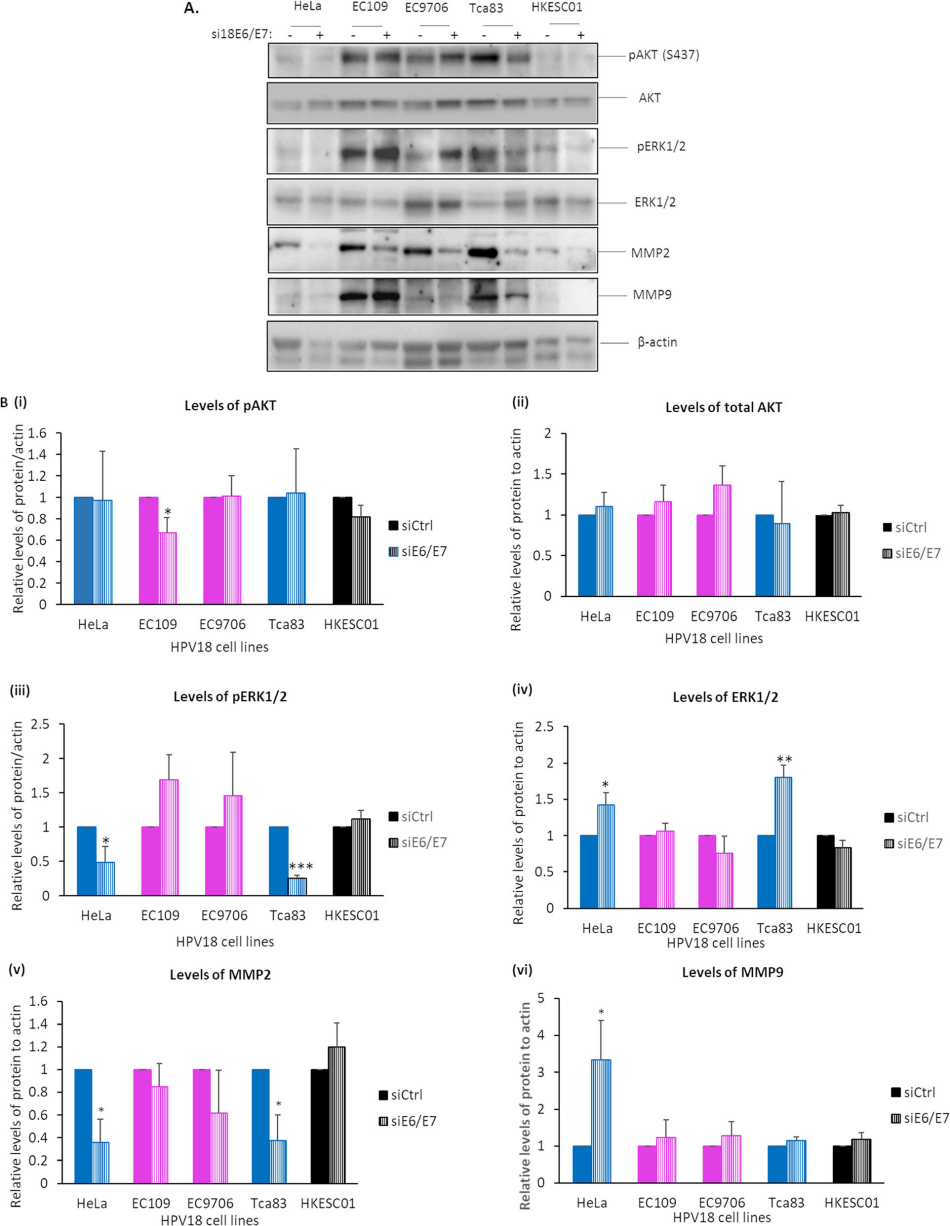
Effects of downregulation of HPV18 E6 and E7 on AKT, ERK1/2, MMP2 and MMP9 activity in EC109, EC9706 and Tca83 cells. These cells were transfected with siRNA against control (−) or against HPV18 E6 and E7 (+). After 72 h, total lysate was collected and the levels of E6 and E7 target proteins were analyzed by Western blotting for the proteins as indicated. HeLa and HKESC01 cells were included as positive and negative controls, respectively. **a.** Representative immunoblots showing levels of AKT phosphorylated at S437 [pAKT(S437)], pan-AKT, ERK1/2 phosphorylated at T202 and Y204 [pERK1/2(T202/Y204)], MMP2 and MMP9. β-actin was included as a loading control. **b.** Bar graphs show quantitation of the levels of target proteins against control in (i) HeLa (blue-colored bars), (ii) EC109 (magenta-colored bars), (iii) EC9706 (magenta-colored bars), (iv) Tca83 (blue-colored bars) and (v) HKESC01 (black-colored bars) cells. Quantitation was performed using ImageJ software and statistical analysis was performed using Prism. Error bars represent mean ± standard deviation (SD) (*n* = 4). (*P < 0.05, **P < 0.01, ***P < 0.001)

It is known that HPV18 oncoproteins can exert their oncogenic properties through targeting AKT [29], extracellular signal-regulated kinase (ERK) [30] and metalloprotease (MMP) [31, 32] pathways in cervical cancer cells, leading to cell survival, proliferation and metastasis. To date, the involvement of HPV18 oncoproteins in perturbing these pathways in esophageal and tongue SCC cell lines has not been clearly defined. This prompted us to look at the levels of AKT, ERK 1/2, MMP2 and MMP9 activities in esophageal (EC109 and EC9706) and tongue (Tca83) cells. In general, we observed a higher basal level of both total and phosphorylated AKT at position S473 [pAKT(S473)], ERK 1/2 phosphorylated at position T202/Y204 [pERK1/2(T202/Y204)], MMP2 and MMP9 in EC109, EC9706 and Tca83 compared to HeLa cells (Fig. 3a). Despite this, we found that Tca83 cells had similar behavior to HeLa cells in targeting ERK and MMP2 pathways, while both EC109 and EC9706 cells were distinct in targeting these pathways through HPV18 oncoproteins.

When HPV18 E6 and E7 in Tca83 cells were depleted using siRNA, we observed significant reduction in pERK1/2 (T202/Y204) and MMP2, together with a significant elevation in ERK1/2 in Tca83 [Fig. 3a, b (iii-v)]. These changes were also observed in HeLa cells. While MMP9 was markedly increased in HeLa cells, no significant change was observed in Tca83 cells [Fig. 3a, b (vi)].

Meanwhile, EC109 and EC9706 cells appeared to be different from HeLa cells. Downregulation of E6 and E7 resulted in a dramatic reduced level of AKT in EC109, but not in the other cells [Fig. 3a, Fig. 3b (i) and (ii)]. In addition, E6 and E7 downregulation had no significant effect on ERK activity, MMP2 and MMP9 levels in EC109 and EC9706. These results revealed that Tca83 had similar behavior to HeLa cells in regulating ERK1/2 activity and MMP2, and both esophageal SCCs were distinct from Tca83 and HeLa cells. Nevertheless, HPV18 oncoproteins appeared to perturb AKT activity in EC109 cells.

### Both Tca83 and HeLa cells require HPV18 oncoproteins to regulate the caspase pathway and proliferate

HeLa cells are addicted to HPV oncoproteins to survive [41], partly through suppression of the caspase pathway [42, 43]. We investigated whether this was reproducible in esophageal (EC109 and EC9706) and tongue (Tca83) cells using afore described siRNA approach to deplete E6 and E7.

**Fig. 4:**
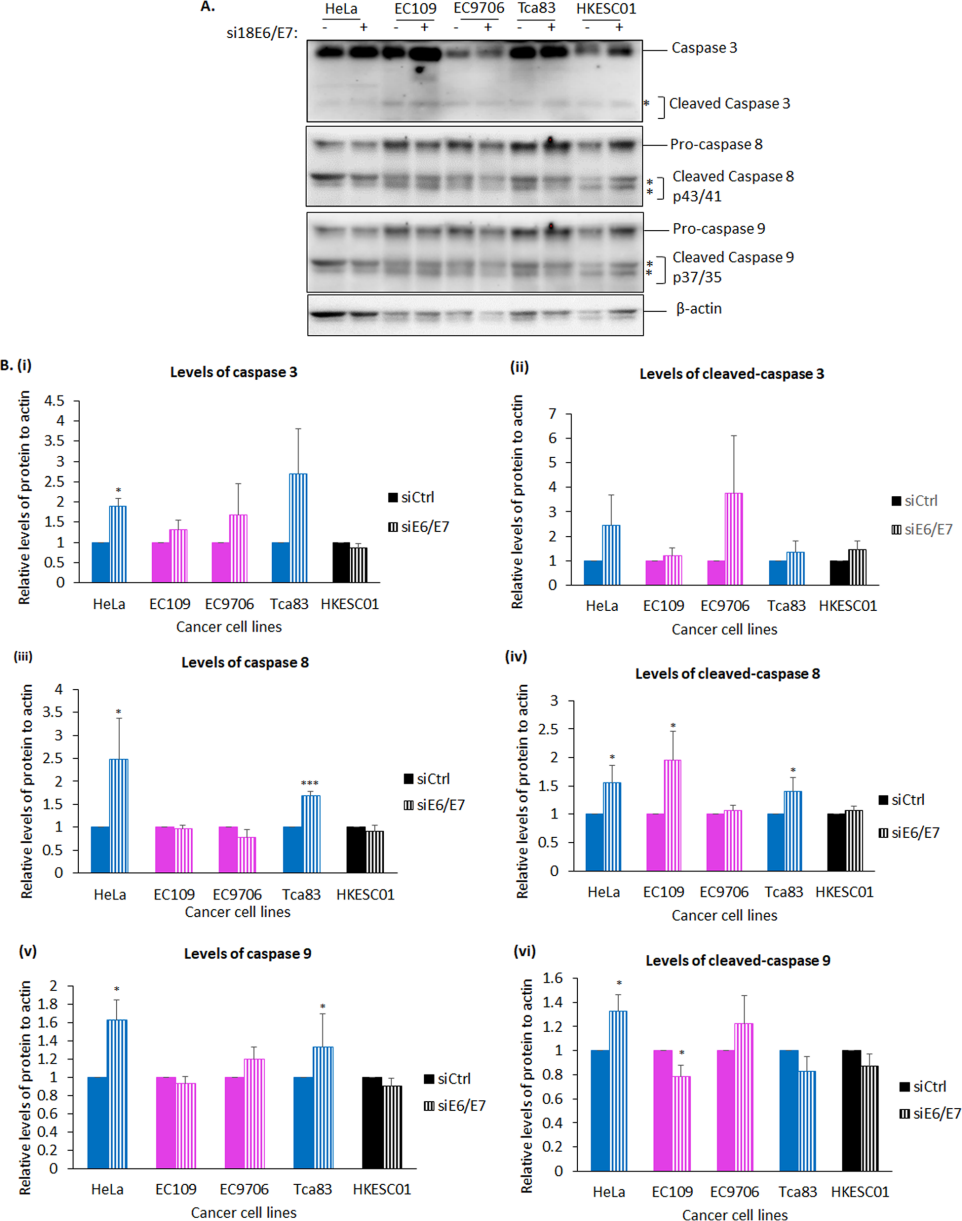
Effects of downregulation of HPV18 E6 and E7 on caspase-dependent apoptotic pathway in EC109, EC9706 and Tca83 cells. These cells were transfected with siRNA against control (−) or against HPV18 E6 and E7 (+). After 72 h, total lysate was collected and the levels of E6 and E7 target proteins was analyzed by Western blotting for the proteins as indicated. HeLa and HKESC01 cells were included as positive and negative controls, respectively. **a.** Representative immunoblots showing the levels of caspase 3, caspase 8, caspase 9 and their respective cleaved forms. β-actin was included as a loading control. **b.** Bar graphs show quantitation of the levels of the (i) caspase 3 and (ii) cleaved caspase 3, (iii) caspase 8 and (iv) cleaved caspase 8, (v) caspase 9 and (vi) cleaved 9, against control in HeLa (blue-colored bars), EC109 (magenta-colored bars), EC9706 (magenta-colored bars), Tca83 (blue-colored bars) and HKESC01 (black-colored bars). Quantitation was performed using ImageJ software and statistical analysis was performed using Prism. Error bars represent mean ± standard deviation (SD) (*n* = 4). (*P < 0.05, **P < 0.01, ***P < 0.001)

We first looked at the levels of initiator (caspases 8 and 9) and effector (caspase 3) caspases. It has been shown that caspase 8 and 9 respond to extracellular apoptotic stimuli [44] and intracellular apoptosomes, respectively. This, in turn, leads to proteolytic and activation of effector caspases, including caspase 3 [45]. Our results showed that ablation of E6 and E7 in HeLa led to a significant increased levels of full length caspases 8, 9 and 3 [Fig. 4a, b (i), (iii) and (v)], as well as cleaved caspases 8 and 9 [Fig. 4a, b (iv) and (vi)]. Interestingly, we also observed increased levels of full length and cleaved caspases 8 and 9 in Tca83 upon depletion of HPV oncoproteins, indicating activation of caspases 8 and 9 [Fig. 4a, b (iii) to (vi)]. However, we did not observe activation of these initiator and effector caspases in EC109 and EC9706 (Fig. 4a and b). These results indicated that, like HeLa cells, E6 and E7 can suppress the caspase cascade in Tca83, but not in EC109 and EC9706.

**Fig. 5:**
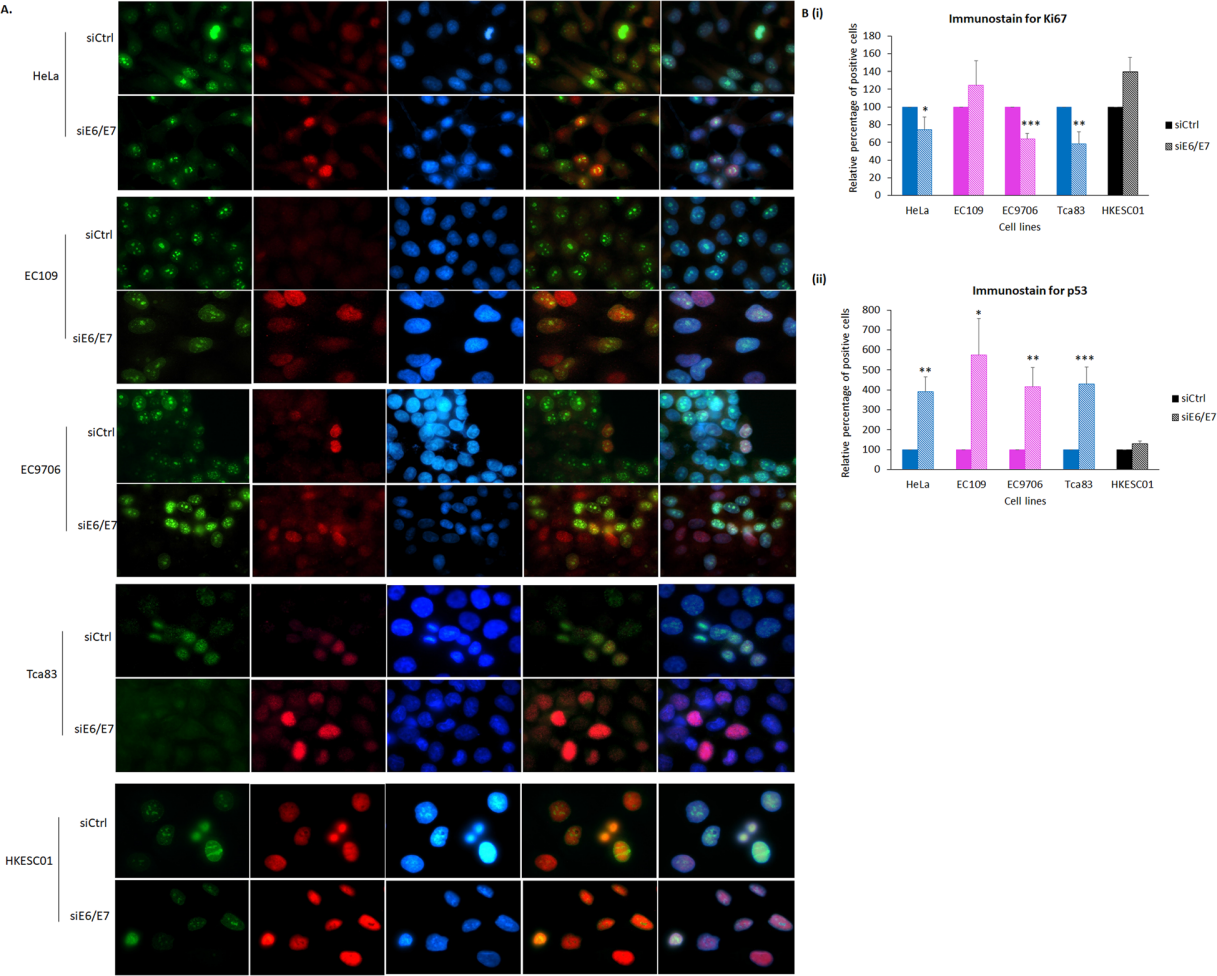
Effects of downregulation of HPV18 E6 and E7 on proliferation of EC109, EC9706 and Tca83 cells. **a.** The cells were transfected with siRNA against control (siCtrl) or against HPV18 E6 and E7 (siE6/E7). After 72 h, cells were fixed and stained with Ki67 (green) and p53 (red). Samples were counterstained with 4′,6-diamidino-2-phenylindole (DAPI) and mounted using ProLong™ Gold Anti-fade. HeLa and HKESC01 cells were included as positive and negative controls, respectively. Images were taken using a fluorescence microscope (Leica) at 1000X. **b.** Images at 400X were obtained from at least 3 independent views per experiment. Cells stained for DAPI, Ki67 and p53 were counted using ImageJ software. Percentage of cells positive for (i) Ki67 and (ii) p53 in siE6/E7 samples were calculated in relative to control (siCtrl). Error bars represent mean ± standard error of mean (SEM) (*n* = 3). (*P < 0.05, **P < 0.01, ***P < 0.001)

We then wanted to know if esophageal and tongue SCC cells are dependent on HPV oncoproteins to proliferate. After E6 and E7 were depleted, we performed immunofluorescence assays to study the levels of Ki67 expression, a commonly used proliferation biomarker for cervical cancer. We also co-stained the cells with p53 as a measure of siRNA efficiency against HPV18 E6 and E7. Consistently, we observed a significant increased level of p53 upon depletion of HPV18 E6 and E7 in all HPV18-positive cells [Fig. 5a and b (ii)]. We observed a significant reduction in Ki67 expression in HeLa, EC9706 and Tca83, but not in EC109 upon ablation of E6 and E7 [Fig. 5a and b (i) and (ii)]. It is worth noting that Ki67 expression was relatively lower in HKESC01 than in other HPV-positive cells. Our results indicated that E6 and E7 promote proliferation of EC9706 and Tca83. Surprisingly, ablation of E6 and E7 was not adequate to initiate activation of caspase pathway in both EC109 and EC9706, as well as did not affect proliferation of EC109.

Overall, our data showed that, alike HeLa cells, Tca83 cells depend on HPV oncoproteins to attenuate initiator caspases and proliferate. In contrast, EC109 and EC9706 cells did not depend on HPV18 E6 and E7 to stimulate apoptosis. However, EC9706 cells require HPV oncoproteins to proliferate.

## Discussion

The fact that high-risk HPV infection is associated with cancers of the uterine cervix, oropharynx, anus, vulvar and penis has been proven beyond doubt. However, the etiological role of HPV in cancers arise from esophageal and tongue remains controversial [15–18]. In this study, we provided for the first time, a comparative molecular analysis among SCC cell lines originated from esophagus, tongue and uterine cervix to delineate their similarities and differences in terms of E6 and E7 transcript expressions and cellular targets.

Since viral integration with loss of E2 transcript is a hallmark of HPV-mediated oncogenesis, we first examined HPV transcription profiles in these cell lines. Our RNA-seq data showed partial expression of HPV genome in esophageal cell lines (EC109 and EC9706), which supports previous observations that integration of HPV genome has occurred [46]. Furthermore, we also observed a similar expression profile in the tongue cell line (Tca83), which suggests that viral integration has occurred. Interestingly, while the expression profiles of HPV genomes were similar among the three types of cancers, the relative ratios of E6, E7 and spliced E6 isoform I (E6^*^I) were different. Both EC109 and EC9706 expressed relatively higher levels of E7 and E6^*^I, while HeLa and Tca83 expressed relatively higher levels of E6. This expression pattern might reflect a functional resemblance of Tca83 to HeLa, and EC109 to EC9706, however, this does not necessarily reflect their respective ability in targeting known cellular proteins.

When we downregulated HPV18 E6 and E7 using siRNA approach, we expected to observe a rescue of E6 and E7 commonly targeted proteins for degradation: p53, p21 and hDlg by E6; pRB and its related pocket proteins, p130 and p107 by E7. In the esophageal and tongue cancer cell lines, E6 appeared to play the expected classical role in downregulating p53 and its downstream target p21 in a similar manner. This is most likely owing to the presence of similar p53 variant containing Arginine residue at codon 72 in all the cell lines examined, which is a form preferably degraded by E6 [39]. However, E6 did not degrade hDlg in esophageal and tongue cancer cell lines. As we did not measure other protein targets, the effect on PDZ proteins like hScribble [24] and MAGIs [23] remains to be clarified.

E7 might target different pathways in esophageal and tongue cancer cells compared to cervical cancer. Classically, pRB appears to be a better E7 degradation target in relative to p130 and p107. Intriguingly, we found that p130 was preferentially targeted by E7 in esophageal cancer cell lines. However, this was not observed in the tongue cancer cell line. This could be partly related to its lower E7 transcript expression than that of HeLa, EC109 and EC9706.

HPV-positive cells require HPV oncoproteins to survive and proliferate, which can occur through perturbation of signaling pathways, including AKT [29], ERK [30], suppression of the caspase cascade [42, 43], and MMPs for migration [31, 32]. AKT is known to possess a pro-survival role [41, 47], while ERK1/2 appears to be a dual-faced protein: anti- or pro-apoptotic kinase [48]. Activation of ERK1/2 was found to either activate or dampen caspase 8 and 9 [49, 50]. Its activation can also activate MMPs [51]. In this study, we found that, consistent with expression patterns of HPV transcripts, tongue cancer cells had similar behavior to cervical cancer cells. In our hand, HPV oncoproteins in these two cells preferentially target the ERK1/2 pathway. This might occur through inactivation of the ERK1/2 upstream protein, mitogen-activated protein/extracellular signal-regulated kinase (MEK), as a result of feedback regulation by ERK1/2 [52]. This might in turn lead to inactivation of initiator caspase 8 and 9, increased proliferation and MMP2-mediated migration.

The role of HPV oncoproteins in EC109 and EC9706 in cellular targeting are indeed distinct from that in HeLa and Tca83. Although these cells share certain degrees of similarity, EC109 and EC9706 can differ from each other in term of HPV18 genome transcripts and subset of cellular proteins targeted by HPV oncoproteins. Both esophageal cancer cell lines do not depend on HPV18 oncoproteins to perturb ERK1/2, caspases and MMPs pathways. The higher basal levels of ERK1/2 and MMPs compared to HeLa cells are sufficient to drive carcinogenesis independent of HPV18 oncoproteins. As both these cells were isolated from different patients, on top of HPV infection, these cells might be exposed to different co-factors, such as environmental factors and genetic predisposition, causing cells to undergo multi-steps carcinogenesis differently. At the molecular level, we found in EC109 that HPV18 oncoproteins can upregulate AKT activity, as reported previously [29], a process essential for cells to resist caspase-dependent apoptosis, proliferate and migrate independent of MMPs. Although EC9706 cells appeared do not require HPV oncoproteins to manipulate signaling and apoptotic pathways examined in this study, they required HPV oncoproteins to proliferate. This could occur through perturbation of transforming growth factor-β1 (TGF-β1) signaling, which is important for epithelial-mesenchymal transition (EMT) of EC9706 [53], and subsequently leading to inactivation of Fas-mediated apoptosis [54] in a caspase-independent fashion. However, this remains unknown and deserves further investigation.

Overall, in line with the model of HPV-mediated cervical carcinogenesis, p53 is the major target of E6 in the esophageal and tongue cancer cell lines examined, whilst p130 is preferentially targeted in esophageal cancer cells. In tongue cancer cells, ERK1/2 and MMP2 pathways appeared to be crucial, but not E7-pRB and AKT pathways. In esophageal cells, a high basal level of AKT, ERK1/2 and MMP activity compared to HPV-null esophageal and HPV-positive cervical cancer cells could be essential for multi-steps cancer progression. Nonetheless, our studies were bound to limitation. In our study, patient-derived cell lines were used to elucidate the role of HPV18 oncoproteins in a monolayer culture system. Physiological relevant models, such as 3-dimensional (3D) culture and animal models should be employed to gain a better understanding on how HPV18 oncoproteins interact with tumour microenvironment and drive cancer progression.

## Conclusion

In conclusion, our findings support that HPV could play an etiological role in esophageal and tongue cancers upon presence of other essential co-factors, such as environmental and genetic predisposition. Yet, the molecular pathways mediated by HPV oncoproteins could be different and are likely determined by tissue specific factors. Further studies focusing on esophageal and tongue cancers are needed to elucidate the full spectrum of HPV-associated cancers, and thus the potential benefits offered by HPV vaccines and treatment.

## Abbreviations

E6*: Spliced E6
EC: Esophageal cancer
EMT: Epithelial-mesenchymal transition
ERK1/2: Extracellular signal-regulated kinase 1/2
FPKM: Fragments per kilobase per million reads mapped
HNC: Head and neck cancer
HPV: Human papillomavirus (HPV)
MEK: Mitogen-activated protein/extracellular signal-regulated kinase
MMPs: Metalloproteases
PAHs: Polycyclic aromatic hydrocarbons
PDZ: PSD95/Dlg/ZO-1
RB: Retinoblastoma
SCC: Squamous cell carcinoma
siRNA: Small interfering RNA
