# Correction to: Human papillomavirus type 18 oncoproteins exert their oncogenicity in esophageal and tongue squamous cell carcinoma cell lines distinctly

**DOI:** 10.1186/s12885-019-6493-4

**Published:** 2019-12-29

**Authors:** Siaw Shi Boon, Zigui Chen, Jintao Li, Karen Y. C. Lee, Liuyang Cai, Rugang Zhong, Paul K. S. Chan

**Affiliations:** 1 Department of Microbiology, Faculty of Medicine, The Chinese University of Hong Kong, Shatin, Hong Kong; 2 Beijing Key Laboratory of Environmental and Viral Oncology, College of Life Science and Bio-engineering, Beijing University of Technology, Beijing, China

**Correction to: BMC Cancer (2019) 19:1211**

**https://doi.org/10.1186/s12885-019-6413-7**

Following publication of the original article [1], the authors reported that during the production process, Table 1 was omitted. Table 1 can be found below. The publishers apologize for this error.

**Table 1:**
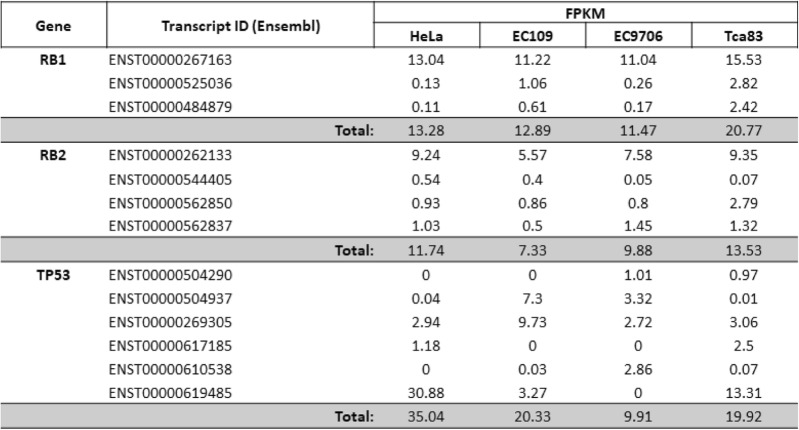
The fragments per kilobase million (FPKM) value of RB1, RB2, and TP53 transcripts in Hela, EC109, EC9706 and Tca83
